# Lung transplantation for diffuse pulmonary arteriovenous malformations associated with juvenile polyposis–hereditary hemorrhagic telangiectasia overlap syndrome: a case report

**DOI:** 10.1186/s44215-024-00183-1

**Published:** 2024-12-27

**Authors:** Taiki Ryo, Daisuke Nakajima, Satoshi Kimura, Hiroshi Date

**Affiliations:** 1 Department of Thoracic Surgery, Kyoto University Graduate School of Medicine, Kyoto, 606-8507 Japan; 2 Department of Anesthesia, Kyoto University Graduate School of Medicine, Kyoto, Japan

**Keywords:** Lung transplantation, Hereditary hemorrhagic telangiectasia, Pulmonary arteriovenous malformations, Juvenile polyposis

## Abstract

**Background:**

Lung transplantation is a viable lifesaving option for patients with diffuse pulmonary arteriovenous malformations (AVMs). We present a case of diffuse pulmonary AVMs associated with juvenile polyposis and hereditary hemorrhagic telangiectasia (JP-HHT) that was successfully managed by lung transplantation.

**Case presentation:**

A 19-year-old woman developed severe hypoxemia due to pulmonary AVMs diagnosed at 4 years of age. She also had epistaxis, hemangioma of the tongue, and numerous polyps in the gastrointestinal tract, leading to the JP-HHT diagnosis. Although she had undergone transcatheter embolization for pulmonary AVMs four times, all lesions became recanalized, and her hypoxemia never improved. She also had hepatic AVMs that did not result in portal hypertension or required any interventions. She underwent bilateral lung transplantation from a brain-dead donor at 3 years after registration. Given that she had severe hypoxemia caused by intrapulmonary shunting, venoarterial extracorporeal membrane oxygenation (V-A ECMO) support was initiated from the femoral vessels under local anesthesia. Then, she was anesthetized and intubated. Peripheral V-A ECMO was switched to central cardiopulmonary bypass during the transplant procedure to prevent persistent hypoxia of the upper body and thromboembolic event due to severe polycythemia. The total graft ischemic time was > 11 h, which resulted in ischemia–reperfusion injury immediately after transplantation. Furthermore, the patient’s postoperative course was complicated by acute cellular rejection and right heart failure due to hepatic AVM progression. She was finally discharged home without oxygen therapy on postoperative day 68. At 1-year post-transplantation, she is currently enjoying college life. However, she still has to undergo periodic endoscopic examinations to monitor her numerous polyps, which are known to carry a risk of cancer development.

**Conclusions:**

Lung transplantation can be a viable treatment option for diffuse pulmonary AVMs in patients with JP-HHT. However, meticulous perioperative management is mandatory to prevent the development of multiple organ disorders.

## Background

Although transcatheter embolization is the first-choice treatment for pulmonary arteriovenous malformations (AVMs) [1], its effectiveness is limited for multiple lesions, and it may cause recurrent AVMs. Lung transplantation can be performed to treat diffuse pulmonary AVMs. However, hereditary hemorrhagic telangiectasia (HHT) is characterized by not only pulmonary AVMs but also recurrent epistaxis, gastrointestinal telangiectasias, and hepatic/cerebral/spinal AVMs. We present a case of diffuse pulmonary AVMs associated with juvenile polyposis–hereditary hemorrhagic telangiectasia (JP-HHT) overlap syndrome that was successfully treated with diseased donor-lung transplantation.

Case presentation.

**Fig. 1:**
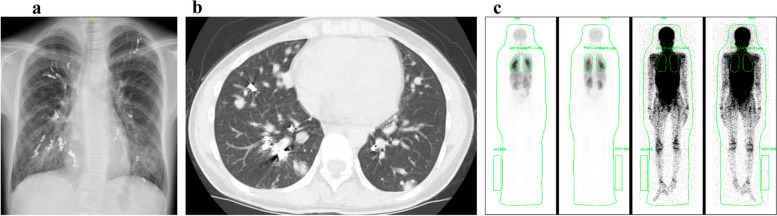
Chest X-ray radiography (**a**) and computed tomography (**b**) showing diffuse pulmonary arteriovenous malformations, which were treated with transcatheter coil embolization. Pulmonary perfusion scintigraphy showing an intrapulmonary shunting rate of 68% (**c**)

**Fig. 2:**
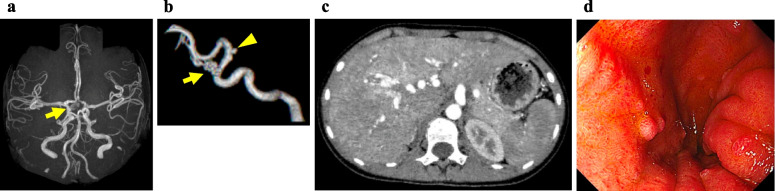
Brain magnetic resonance angiography showing two cerebral aneurysms, which are located at the right internal carotid–ophthalmic (arrow) and right internal carotid–anterior choroidal (arrowhead) arteries (**a**, **b**). Contrast-enhanced abdominal computed tomography showing hepatic arteriovenous malformations (**c**). Gastroenterological endoscopy showed diffuse polyps in the duodenum (**d**)

A 19-year-old woman developed severe hypoxemia due to pulmonary AVMs diagnosed at 4 years of age. At 13 years of age, she was then diagnosed with HHT based on the physical findings of recurrent epistaxis and hemangioma of the tongue. A SMAD4 germline mutation was detected in the patient, despite not having a family history of HHT. She underwent transcatheter embolization four times to treat the pulmonary AVMs, but the treatment failed, and all lesions became recanalized (Fig. 1a, b). Furthermore, numerous polyps were seen in the duodenum and colon; she was then finally diagnosed with JP-HHT. At 14 years of age, she was referred to our hospital for possible lung transplantation. She had remarkable shortness of breath with a modified Medical Research Council (mMRC) grade of 3. Her 6-min walking distance was only 180 m on 2 L/min of nasal oxygen. Physical examination revealed cyanosis and clubbed fingers due to severe hypoxemia (PaO_2_: 38 Torr on 2 L/min of nasal oxygen). Blood tests showed significant polycythemia (hemoglobin: 22.1 g/dL). Pulmonary perfusion scintigraphy showed a right-to-left shunting rate of 68% (Fig. 1c). There were several concerns regarding the indication for lung transplantation. First, she also developed other organ disorders associated with JP-HHT. She had two cerebral aneurysms: one was 7.9 mm in diameter and located at the right internal carotid–ophthalmic artery (Fig. 2a), which was treated by coil embolization, and the other was 2 mm in diameter and localized in the right internal carotid–anterior choroidal artery (Fig. 2b), which required no intervention. Additionally, contrast-enhanced abdominal computed tomography showed diffuse hepatic AVMs (Fig. 2c), which was not considered a contraindication for lung transplantation because of the absence of complications, such as biliary obstruction and portal hypertension. Second, an upper gastrointestinal endoscopy with colonoscopy revealed JP (Fig. 2d), which was known to be a risk factor of gastrointestinal and colon cancers. Therefore, periodical endoscopic examinations were definitely required for the management of JP. Despite the concerns on the other organ lesions with JP-HHT, none required any intervention during the lung transplant evaluation. Finally, she was registered in the Japan Organ Transplant Network, and a brain-dead donor lung was allocated for her at 3 years after registration.

Subsequently, she underwent bilateral lung transplantation. Owing to the significant hypoxemia due to shunting with a rate of 68% and the risk of pulmonary AVM rupture due to positive-pressure ventilation, venoarterial extracorporeal membrane oxygenation (V-A ECMO) support was initiated by femoral arterial and venous cannulations under local anesthesia. Then, general anesthesia was administered. Given her history of recurrent epistaxis and hemangioma of the tongue, she was intubated with a single-lumen endotracheal tube, instead of a double-lumen endotracheal tube, to minimize the risk of oral bleeding caused by endotracheal intubation. Following the clamshell thoracotomy, peripheral V-A ECMO was switched to cardiopulmonary bypass (CPB) via ascending aorta and bicaval cannulations to avoid persistent hypoxia of the upper body and the development of a thrombotic event due to severe polycythemia. Then, ventilation was completely stopped. After the bilateral pneumonectomies, the right and left donor lungs were sequentially implanted. She was successfully weaned off CPB after reperfusion. The total ischemic time of the implanted lung grafts was > 11 h, complicating direct chest closure due to pulmonary edema caused by an ischemia–reperfusion injury. Expanded polytetrafluoroethylene sheets without rib approximation were used to temporarily close the chest. The patient had severe adhesions and neovascularization between the lungs and chest wall and required CPB support, which caused massive and uncontrolled bleeding during the transplant procedures. The operative time was 544 min, and the amounts of blood loss and blood transfusion were 5000 and 4280 mL, respectively.

**Fig. 3:**
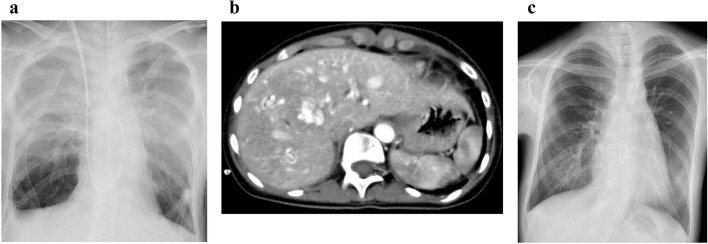
Postoperative chest X-ray radiography showed bilateral infiltrates due to ischemia–reperfusion injury that developed immediately after lung transplantation (**a**). Contrast-enhanced abdominal computed tomography showed the progression of hepatic arteriovenous malformations on postoperative day 30 (**b**). Chest X-ray radiography at discharge (**c**)

Three major adverse events occurred after transplantation. First, primary graft dysfunction (PGD) due to ischemia–reperfusion injury was observed post-transplantation (PGD grades 3, 1, and 2 at 24, 48, and 72 h after transplantation, respectively) (Fig. 3a). Thereafter, the patient’s pulmonary edema gradually improved, and her chest was finally closed on postoperative day (POD) 5. Second, she experienced acute cellular rejection on POD 30, which was successfully treated with methylprednisolone pulse therapy (500 mg/day) for 3 days. Third, she developed severe right-heart failure associated with exacerbation of diffuse hepatic AVMs (Fig. 3b). Echocardiogram showed hypercontraction of left ventricle and prominent enlargement of the inferior vena cava after transplantation. Although coil embolization of the liver AVMs was not feasible due to their significant multiplicity and stable liver function, the right-heart failure was successfully managed by treatment with diuretics (furosemide and tolvaptan), and she was successfully weaned from the ventilator on POD 36. No neurological disorders suggesting rupture of cerebral aneurysms were observed. Finally, after showing improved exercise tolerance, with her 6-min walk distance being 350 m with a minimum peripheral oxygen saturation (SpO_2_) of 96% on room air (Fig. 3c), she was discharged home without oxygen therapy on POD 68. At 1 year after transplantation, she is currently enjoying college life, with favorable respiratory function (vital capacity: 2.60 L; forced expiratory volume in 1 s: 2.21 L) and oxygen saturation levels (SpO_2_ > 98% on room air).

## Discussion and conclusions

Transcatheter embolization has a viable role in pulmonary AVM treatment [1]. However, this intervention can lead to poor outcomes because AVM recanalization can occur in multiple lesions. Therefore, the only radical treatment procedure for such diffuse pulmonary AVMs is lung transplantation [2–4]. As soon as the effect of transcatheter embolization cannot be expected any more, the patient should be referred to transplant center for listing for lung transplantation. However, besides pulmonary AVMs, HHT is associated with various conditions in other organs. Therefore, careful evaluation of the indication of lung transplantation for pulmonary AVMs should be performed in HHT patients, and they should be meticulously monitored before and after transplantation.

Approximately, 80% of HHT cases involve ENG and ALK1 mutations, whereas SMAD4 mutation only occurs in 2% of cases. The frequency of major conditions, including epistaxis, telangiectasia, and pulmonary/brain/liver AVMs, does not significantly differ among patients with different HHT causative genes. However, HHT patients with the SMAD4 mutation more frequently have diffuse gastrointestinal polyps as compared to those with other gene mutations, increasing their risk of developing gastrointestinal and colon cancers [5]. Early onset of such cancers is reported in approximately 20% of those cases [6]. Following lung transplantation, the lifelong administration of immunosuppressive agents, including tacrolimus, mycophenolate mofetil, and prednisolone, could worsen this risk. Hence, periodic endoscopic examinations are required after transplantation.

There are several important points for the induction of general anesthesia in patients with HHT. In the present case, the patient had recurrent epistaxis and hemangioma of the tongue, and bleeding from telangiectasias in the oral cavity and nasopharynx was the primary concern in the anesthetic management [7]. Therefore, we performed the single-lumen endotracheal intubation, which was considered to be easier and safer than the complicated double-lumen endotracheal intubation. Second, positive-pressure ventilation increases the risk of rupture of pulmonary AVMs and cerebral aneurysms [8]. Additionally, severe hypoxia due to intrapulmonary shunting could enhance pulmonary vasoconstriction, leading to an increase in pulmonary arterial pressure [9], which might also enhance the rupture of pulmonary AVMs. Therefore, V-A ECMO support was initiated under local anesthesia before endotracheal intubation, as it could reduce the positive airway pressure in mechanical ventilation. Third, the patient required a central CPB using full heparinization during the transplant procedure. The patient was intubated with a single-lumen endotracheal tube, instead of a double-lumen endotracheal tube, to minimize the risk of oral bleeding caused by endotracheal intubation. This indicates that we needed to completely stop ventilation during the transplant procedures. Therefore, we switched the peripheral VA-ECMO to central CPB in order to provide enough oxygenated blood flow for the patient, while mechanical ventilation was completely interrupted.

In our case, the patient had mild liver AVMs that did not lead to portal hypertension and liver cirrhosis at the time of registration. However, these AVMs progressed while she was waiting for 3 years for her transplantation. The diffuse liver AVM progression could cause high-output heart failure with an increased risk of pleural effusion and prominent enlargement of the inferior vena cava after lung transplantation [10], which could be finally controlled by the administration of large doses of diuretics. In Japan, the mean waiting period of deceased-donor lung transplantation is currently 32 months after the registration [4], indicating that the AVMs in other organs should be carefully monitored while waiting for the allocation of brain-dead donor lungs.

In conclusion, lung transplantation is a feasible treatment procedure for diffuse pulmonary AVMs associated with JP-HHT. However, careful evaluation of the indication for lung transplantation and meticulous perioperative management is required to prevent the development of severe posttransplantation complications due to various disorders in other organs.

## Data Availability

Data are available upon request due to privacy/ethical restrictions.

## Abbreviations

AVMs: Arteriovenous malformations
HHT: Hereditary hemorrhagic telangiectasia
JP: Juvenile polyposis
mMRC: Modified medical research council
CT: Computed tomography
V-A ECMO: Venoarterial extracorporeal membrane oxygenation
CPB: Cardiopulmonary bypass
POD: Postoperative day
